# Nitrification Is a Primary Driver of Nitrous Oxide Production in Laboratory Microcosms from Different Land-Use Soils

**DOI:** 10.3389/fmicb.2016.01373

**Published:** 2016-09-09

**Authors:** Rui Liu, Hangwei Hu, Helen Suter, Helen L. Hayden, Jizheng He, Pauline Mele, Deli Chen

**Affiliations:** ^1^Faculty of Veterinary and Agricultural Sciences, The University of MelbourneMelbourne, VIC, Australia; ^2^Department of Economic Development, Jobs, Transport and ResourcesMelbourne, VIC, Australia

**Keywords:** nitrification, AOA, AOB, bacteria, archaea, land-use management

## Abstract

Most studies on soil N_2_O emissions have focused either on the quantifying of agricultural N_2_O fluxes or on the effect of environmental factors on N_2_O emissions. However, very limited information is available on how land-use will affect N_2_O production, and nitrifiers involved in N_2_O emissions in agricultural soil ecosystems. Therefore, this study aimed at evaluating the relative importance of nitrification and denitrification to N_2_O emissions from different land-use soils and identifying the potential underlying microbial mechanisms. A ^15^N-tracing experiment was conducted under controlled laboratory conditions on four agricultural soils collected from different land-use. We measured N_2_O fluxes, nitrate (${\text{NO}}_{3}^{-}$), and ammonium (${\text{NH}}_{4}^{+}$) concentration and ^15^N_2_O, ^15^${\text{NO}}_{3}^{-}$, and ^15^${\text{NH}}_{4}^{+}$ enrichment during the incubation. Quantitative PCR was used to quantify ammonia-oxidizing archaea (AOA) and ammonia-oxidizing bacteria (AOB). Our results showed that nitrification was the main contributor to N_2_O production in soils from sugarcane, dairy pasture and cereal cropping systems, while denitrification played a major role in N_2_O production in the vegetable soil under the experimental conditions. Nitrification contributed to 96.7% of the N_2_O emissions in sugarcane soil followed by 71.3% in the cereal cropping soil and 70.9% in the dairy pasture soil, while only around 20.0% of N_2_O was produced from nitrification in vegetable soil. The proportion of nitrified nitrogen as N_2_O (P_N2O_-value) varied across different soils, with the highest P_N2O_-value (0.26‰) found in the cereal cropping soil, which was around 10 times higher than that in other three systems. AOA were the abundant ammonia oxidizers, and were significantly correlated to N_2_O emitted from nitrification in the sugarcane soil, while AOB were significantly correlated with N_2_O emitted from nitrification in the cereal cropping soil. Our findings suggested that soil type and land-use might have strongly affected the relative contribution of nitrification and denitrification to N_2_O production from agricultural soils.

## Introduction

Ammonium-based fertilizers are extensively used in agricultural practices to meet the food demand for the increasing human population, which has resulted in an increase in atmospheric N_2_O concentrations (Galloway et al., 2008; Davidson, 2009). Globally, natural and anthropogenic N_2_O sources are primarily dominated by emissions from soil ecosystems, comprising approximately 65% of the total N_2_O emissions (IPCC, 2007). In Australia, agriculture is the second largest greenhouse gas (GHG) source, accounting for 16% of total GHG emissions, 19% of which could be attributed to N_2_O emitted from agricultural soils (Australian Greenhouse Office, 2001).

The emission of N_2_O is the result of multiple biological pathways, such as nitrification (autotrophic and heterotrophic), denitrification, dissimilatory nitrate reduction to ammonium (DNRA), nitrifer denitrification, and non-biological chemodenitrification (Wrage et al., 2001; Butterbach-Bahl et al., 2013; Hu et al., 2015a; Zhang et al., 2015), but is particularly dominated by nitrification and denitrification (Davidson et al., 1986; Stevens et al., 1997; Hu et al., 2015a). As multiple pathways involved in N_2_O production and N_2_O consumption occur simultaneously in different micro-environments in the same soil, a great challenge exists in allocating their relative contributions. Nitrification inhibitors and isotope signature techniques are commonly utilized to separate N_2_O-producing and -reducing pathways (Zhang et al., 2009). Stable isotope enrichment approaches have been developed to identify N_2_O sources following the application of ^15^N-labeled fertilizers in short-term experiments, through the measurement of ^15^N enrichment in N_2_O and mineral N pools (Baggs, 2008). Application of ^15^N labeled ${\text{NH}}_{4}^{+}$ and ${\text{NO}}_{3}^{-}$ enables the source of fertilizer-derived ^15^N-N_2_O to be determined. Generally, denitrification-derived N_2_O is quantified following the supply of ^15^${\text{NO}}_{3}^{-}$, while nitrification derived N_2_O is measured following the supply of ^15^${\text{NH}}_{4}^{+}$ (Baggs, 2008). The reduction of N_2_O to N_2_ can also be quantified by determining ^15^N in N_2_ after the supply of ^15^${\text{NO}}_{3}^{-}$ (Stevens and Laughlin, 1998). For example, applications of ^14^${\text{NH}}_{4}^{15}$NO_3_ and ^15^${\text{NH}}_{4}^{14}$NO_3_ have been used to determine the relative contributions of nitrification and denitrification to N_2_O production (Baggs and Blum, 2004).

Agricultural practice, climatic conditions and soil properties all influence N_2_O emission from soil. These include soil moisture and temperature (Livesley et al., 2008), aeration, ammonium, and nitrate concentration (Jørgensen and Elberling, 2012), and pH (Mørkved et al., 2007). Soil water content is one of the predominant factors regulating N_2_O emission from soils. Increasing soil water content due to wetting-up events such as irrigation and rainfall can stimulate nitrification and denitrification (Hu et al., 2015b), and can promote N_2_O production (Hofstra and Bouwman, 2005). N_2_O emission has been found to be highly correlated with water filled pore space (WFPS), with the highest emission under 70% WFPS coming from both nitrification (35–53%) and denitrification (44–58%) pathways in an intensively managed calcareous Fluvo-aquic soil (Huang et al., 2014). The favorable conditions for N_2_O production from nitrification occur within the range of 30–70% WFPS (Hu et al., 2015a), whereas denitrification dominates N_2_O production in wet soils with >80–90% WFPS (Braker and Conrad, 2011; Huang et al., 2014).

To date, most studies on soil N_2_O emissions have focused either on the quantification of agricultural N_2_O fluxes (Reay et al., 2012) or on the effect of environmental factors on N_2_O fluxes (Cantarel et al., 2011). However, very limited information is available on how land-use will affect the relative contributions of nitrification and denitrification to N_2_O production, the nitrified N lost as N_2_O, and the underlying microbial mechanisms in agricultural soil ecosystems.

It has been widely accepted that two groups of ammonia oxidizers, ammonia-oxidizing archaea (AOA), and ammonia-oxidizing bacteria (AOB) are responsible for the first step of nitrification (oxidation of NH_3_ to ${\text{NO}}_{2}^{-}$; Di et al., 2009, 2010; Gubry-Rangin et al., 2010), and the two groups are typically profiled using functional *amo*A gene encoding the alpha sub-unit of ammonia mono-oxygenase (AMO), the key enzyme for ammonia oxidation. The bacterial and archaeal *amo*A genes can be distinguished by their sequences. The conversion of ${\text{NO}}_{2}^{-}$ to ${\text{NO}}_{3}^{-}$, is regulated by nitrite oxidoreductase which is encoded by the functional *nxrB* gene (Freitag et al., 1987). Until recently, AOB were believed to be the only microbes active in nitrification, however ammonia-oxidizing archaea activity in soils has been reported based on *in situ* expression of archaeal *amo*A genes (Treusch et al., 2005; Leininger et al., 2006; Offre et al., 2009). It has been revealed that AOA can also be present in large numbers in terrestrial environments (He et al., 2007; Shen et al., 2008). More recently, the complete oxidation of ammonia to nitrate in one organism (complete ammonia oxidation; comammox) has been reported by Daims et al. (2015) and van Kessel et al. (2015).

Land-use and land management appears a very important factor affecting microbial communities in soils. For instance, Morimoto et al. (2011) reported that land-use types affected the abundances of AOA and AOB and the nitrification activity. Research across different soils in Australia has also revealed that changes in soil variables due to different land-use can strongly influence the abundance of AOB *amo*A gene (Hayden et al., 2010). Increasing evidence has also reported that AOA and AOB can produce N_2_O (Santoro et al., 2011; Stieglmeier et al., 2014; Kozlowski et al., 2016), but their contributions to soil N_2_O emissions in agricultural ecosystems with different land-use and the relevant microbial pathways remain unclear. Therefore, it is necessary to improve the understanding of N_2_O formation and quantify the contribution of different pathways and verify whether land-use is a key factor to influence N_2_O emissions and AOA and AOB function in nitrification.

This study used ^15^N tracer technique to separate nitrification and denitrification to (i)determine the contribution of nitrification and denitrification to nitrous oxide production in laboratory microcosms using ^15^N isotope tracer method; and (ii) quantify the abundance of AOA and AOB in experimental microcosms. We hypothesized that: (i) the relative contribution of nitrification and denitrification changes with different agricultural soils and (ii) the relationship between AOA/AOB populations and N_2_O emission is affected by different agricultural soils.

## Materials and methods

### Soil collection and physicochemical measurement

Soil samples used in this study were collected from four different agricultural sites across Australia: sugarcane at Bundaberg, QLD (24°57′S, 152°20′E), vegetable at Boneo, VIC (38°24′S, 144°53′E), dairy pasture at Longworry, VIC (38°08′S, 145°43′E) and cereal cropping at Hamilton, VIC (38°19′S, 142°42′E). At each site, 10 replicate samples of the top soils (0–10 cm) were collected, thoroughly homogenized, and transported on ice to the laboratory. The fresh soils were air-dried, and remaining roots and leaf pieces were removed with tweezers. Air-dried soils were ground and sieved through a 2.0 mm mesh prior to establishment of the microcosm incubation. Soil moisture contents were determined by oven-drying three subsamples (10 g of air-dried soil) at 105°C for 48 h. Soil pH (1:5 soil/water), texture (sieve and hydrometer procedures), total carbon (Dumas method) and other soil properties are demonstrated in Table 1.

**Table 1:** **Field site description and basic characteristics of soils used in this study**.

**Land-use**	**Site name**	**Climate**	**Texture**	**Clay**	**Sand %**	**Silt**	**pH (H_2_O)**	**NH_4_-N**	**NO_3_-N**	**TC %**	**TN %**
								**mg N kg^−1^ soil**			
Sugarcane	Bundaberg, QLD	Subtropical	Sand	5	90	5	6.0	2.6	8.8	1.2	0.06
Vegetable	Beneo, VIC	Temperate	Sand	1	91	8	7.8	1.1	19	0.8	0.08
Dairy pasture	Longworry, QLD	Tropical	Clay loam	4	75	21	4.8	16	47	9.3	0.8
Cropping	Hamilton, VIC	Temperate	loam	10	61	29	7.0	5.1	10	ND	ND

### Soil microcosm incubation

Soil microcosms were established in 500 ml vials containing 60 g of soils (oven-dry equivalent). Soil microcosms were pre-incubated at 25°C for 3 weeks to stabilize the soil indigenous microbial communities and to minimize effects associated with wetting events. Soil moisture contents during pre-incubation were adjusted to below 50% of the WFPS during the incubation (Linn and Doran, 1984). After pre-incubation, 2 ml of distilled water with or without fertilizers was applied to each vial to reach the targeted 50% WFPS and fertilizer levels. Two sets of treatments were established in four replicates with addition of 100 mg ${\text{NH}}_{4}^{+}$-N and 50 mg ${\text{NO}}_{3}^{-}$-N kg^−1^ soil: (1) ^15^NH_4_Cl (at 10 atom% ^15^N excess) + KNO_3_; and (2) NH_4_Cl + K^15^NO_3_ (at 10 atom% ^15^N excess). Aerobic conditions and soil moisture contents in the vials were maintained every 3 days by opening microcosms for aeration and water replenishment. Soil microcosms were incubated at 25°C in the dark for 3 weeks.

### Gas sampling and analysis

Gas samples (20 ml) for N_2_O and CO_2_ analysis were taken from the headspace of 500 ml vials on days 0, 4, 7, 12, and 15 after fertilizer application. Gas samples (60 ml) for the analysis of fertilizer-derived ^15^N_2_O were taken at 72 h sampling time after vials closure on days 0, 7, and 15. The four replicate gas samples (20 ml) were collected from the 500 ml vials using gas tight syringes at 0, 8, 24, 48, and 72 h for each sampling day. A preliminary test was done before this work commencement, and found out the most suitable five gas collection time points at each collection day to calculate N_2_O production rate. Before gas collection, 20/60 ml compressed zero air were injected into 500 ml vials to keep the pressure in the vials and then collected 20 ml gas samples into the pre-evacuated exetainers (Exetainer®, Labco Ltd., Lampeter, Ceredigion, UK). The 20 ml gas samples were analyzed for concentrations of N_2_O and CO_2_ by gas chromatography (GC, Agilent 7890). Gas samples (60 ml) for the analysis of fertilizer-derived ^15^N_2_O were taken on days 0, 7, and 15 were analyzed for ^15^N enrichment in N_2_O by Isotope Ratio Mass Spectrometry (IRMS, Hydra 20–20, SerCon, Crewe, UK).

### Soil sampling and analysis

Soils were destructively sampled for mineral nitrogen measurements and isotope measurements on days 0, 7, and 15 immediately after gas sampling. There were four replicates at each sampling day. Subsamples of 2 g soil were collected for soil DNA extraction, and 50 g of soil in the 500 ml vials was shaken with 250 ml 2M KCl (1:5 ratio soil:KCl solution) for 1 h at 200 rpm at room temperature, and the supernatant was filtered through a qualitative Whatman No. 42 filter paper. The extracts (30 ml) were stored at −20°C prior to analysis of ${\text{NH}}_{4}^{+}$-N and ${\text{NO}}_{3}^{-}$-N on a segmented-flow analyser (Skalar SAN++, Breda, Holland). The ^15^N enrichment of ${\text{NH}}_{4}^{+}$ and ${\text{NO}}_{3}^{-}$ was determined by a micro-diffusion method as reported by Saghir et al. (1993), with the modification that an acidified filter paper disc (Whatman No. 41) was used instead of the petri dish of acid to absorb NH_3_ and analysis by the Isotope Ratio Mass Spectrometer (Hydra 20–20, Sercon, Crewe, UK).

### Soil DNA extraction and quantitative PCR (qPCR)

The Power Soil DNA Isolation kit (MO BIO Laboratories Inc., Carlsbad, CA, USA) was used for DNA extraction from 0.25 g of soils collected on days 0, 7, and 15 following the manufacturer's instructions. The quantity and quality of the extracted DNA were assessed using a NanoDrop ND2000c spectrophotometer (NanoDrop Technologies, Wilmington, DE, USA) and checked on the 1% agarose gel. The AOA and AOB *amo*A gene copy numbers were quantified from triplicate samples using qPCR with the primer sets Arch-amoAF/Arch-amoAR (Francis et al., 2005) and amoA1F/amoA2R (Rotthauwe et al., 1997), respectively. Each qPCR reaction for AOA was performed in a 20 μl volume containing 10 μl SensiFAST SYBR No-ROX reagent (Bioline, Sydney, Australia), 0.5 μM of each primer, and 2 μl of 10-fold dilution DNA template (1–10 ng). Each qPCR reaction for AOB was performed in a 10 μl volume containing 5 μl iTaq Universal SYBR Green Supermix (Bio-Rad Laboratories, USA), 0.6 μM of each primer, and 2 μl of 10-fold dilution DNA template (1–10 ng). Amplification conditions for both AOA and AOB were as follows: 95°C for 3 mins, 40 cycles of 5 s at 95°C, 30 s at 60°C, and 45 s at 72°C. A known copy number of plasmid DNA for AOA or AOB was used to create a standard curve. For all assays, qPCR efficiency was 92.5–98.7% and *r*^2^ was 0.96–0.99.

### Calculations

N_2_O fluxes were calculated according to the following equation:

$$\begin{array}{l} \text{F}=\rho \times \frac{V}{A}\times \frac{\Delta c}{\Delta t}\times \frac{273}{273+T} \end{array}$$

where F is the gas flux in μg N_2_O-N cm^2^ d^−1^, ρ represents the density of N_2_O under the standard state (g ml^−1^), *V* is the volume of the head space (ml), *A* is the area of the vial (cm^2^), $\frac{\Delta c}{\Delta t}$ is the change in gas concentration per unit of time in ppm d^−1^, and *T* is the air temperature within the vial (°K).

The gross nitrification rate was determined by the ^15^N dilution technique (Kirkham and Bartholomew, 1954; Barraclough and Puri, 1995).

The relative contribution by denitrification (Cd) and nitrification (Cn) to N_2_O production was calculated using the method by Stevens et al. (1997) following the equation:

$$\begin{array}{l} \text{Cd}=\left({\text{a}}_{\text{N}2\text{O}}-{\text{a}}_{\text{NH}4}\right)/\left({\text{a}}_{\text{NO}3}-{\text{a}}_{\text{NH}4}\right);\text{Cn}=1-\text{Cd} \end{array}$$

where a_N2O_ is the ^15^N atom% enrichment of N_2_O, a_NO3_ is the ^15^N atom% enrichment in ${\text{NO}}_{3}^{-}$ pool, and a_NH4_ is the ^15^N atom% enrichment in ${\text{NH}}_{4}^{+}$ pool. Based on Stevens et al. (1997), the relative contribution of nitrification and denitrification to N_2_O emission was calculated from the ^15^N-NO_3_ treatment.

$$\begin{array}{l} {\text{N}}_{2}\text{O production from nitrification }({\text{N}}_{2}{\text{O}}_{\text{n}})\text{ was calculated as}:\\ {\text{ N}}_{2}{\text{O}}_{\text{n}}=\text{Cn}\times {\text{N}}_{2}{\text{O}}_{\text{T}}\\ {\text{N}}_{\text{2}}\text{O production from denitrification }({\text{N}}_{\text{2}}{\text{O}}_{\text{d}})\text{ was calculated as}:\\ {\text{ N}}_{\text{2}}{\text{O}}_{\text{d}}{\text{ = Cd×N}}_{\text{2}}{\text{O}}_{\text{T}}\\ {\text{The proportion of nitrified N emitted as N}}_{\text{2}}{\text{O (P}}_{\text{N2O}}\text{) was}\\ {\text{ calculated as: P}}_{\text{N2O}}{\text{ = N}}_{\text{2}}{\text{O}}_{\text{n}}{\text{/NO}}_{3}^{-} \end{array}$$

Where ${\text{NO}}_{3}^{-}$ is produced through nitrification.

### Statistical analyses

Data were analyzed using SPSS 19 and means were compared using one-way analysis of variance (ANOVA) between treatments to test the variance with a level of significance of *p* < 0.05. Spearman correlation analysis was performed to test the relationships between N_2_O_n_ and the abundances of AOA and AOB.

## Results

### Characteristics of soils used in this study

In this study, the examined soil physical and chemical properties highly varied across different land-use (Table 1). All soils except the vegetable soil were acidic (pH ≤ 6). The dairy pasture soil had the highest total C content (9.3%), while the vegetable soil the lowest (0.8%). The same trends for the total N contents were observed. The inorganic nitrogen was dominated by ${\text{NO}}_{3}^{-}$-N ranging from 8.8 to 47 mg kg^−1^ soil, with the highest value recorded in the dairy pasture soil. Sugarcane and vegetable soils had sandy texture, but dairy pasture soil was clay loam and cropping soil was loam.

### N_2_O production rates

**Figure 1:**
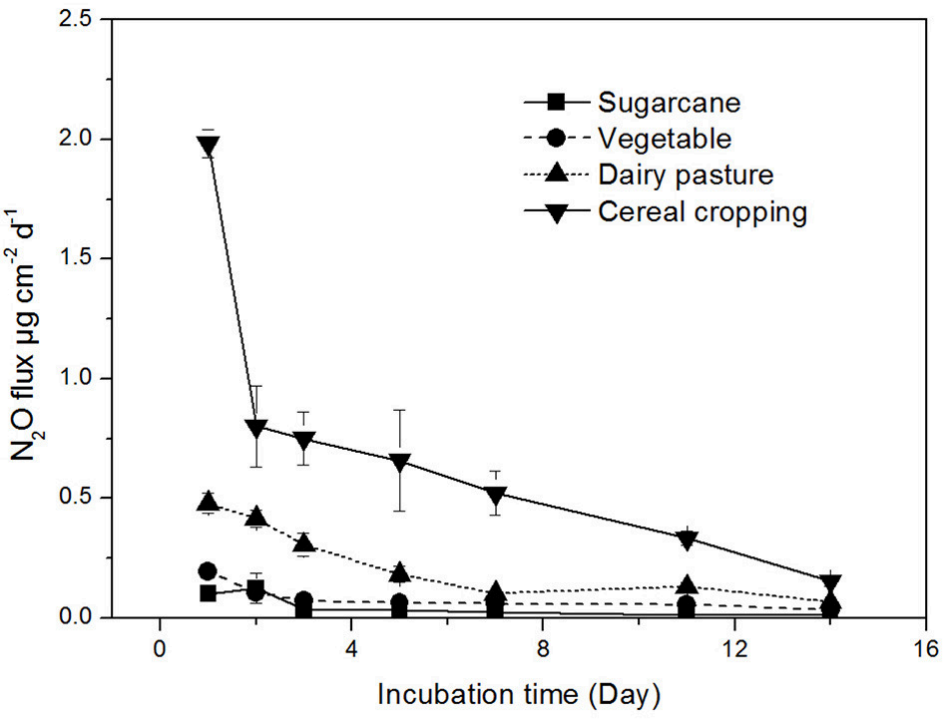
**Soil N_2_O production rates from different agricultural soils**. Error bars represent standard error.

The N_2_O production rates were found to be highly variable across different land-use in agricultural soils (Figure 1). The highest N_2_O production rate was recorded in the cereal cropping soil (average 1.98 μg N_2_O-N cm^−2^ d^−1^), which was significantly higher than those in the sugarcane soil (0.12 μg N_2_O-N cm^−2^ d^−1^), vegetable soil (0.20 μg N_2_O-N cm^−2^ d^−1^), and dairy pasture soil (0.48 μg N_2_O-N cm^−2^ d^−1^). The N_2_O flux continuously decreased throughout the incubation period in the cereal cropping soil, while in the sugarcane, dairy pasture, and vegetable soils, N_2_O production rates stabilized after 7 days of incubation (Figure 1). The soils with higher total N contents tended to have higher N_2_O production rates.

### N_2_O sources in different agricultural soils

**Figure 2:**
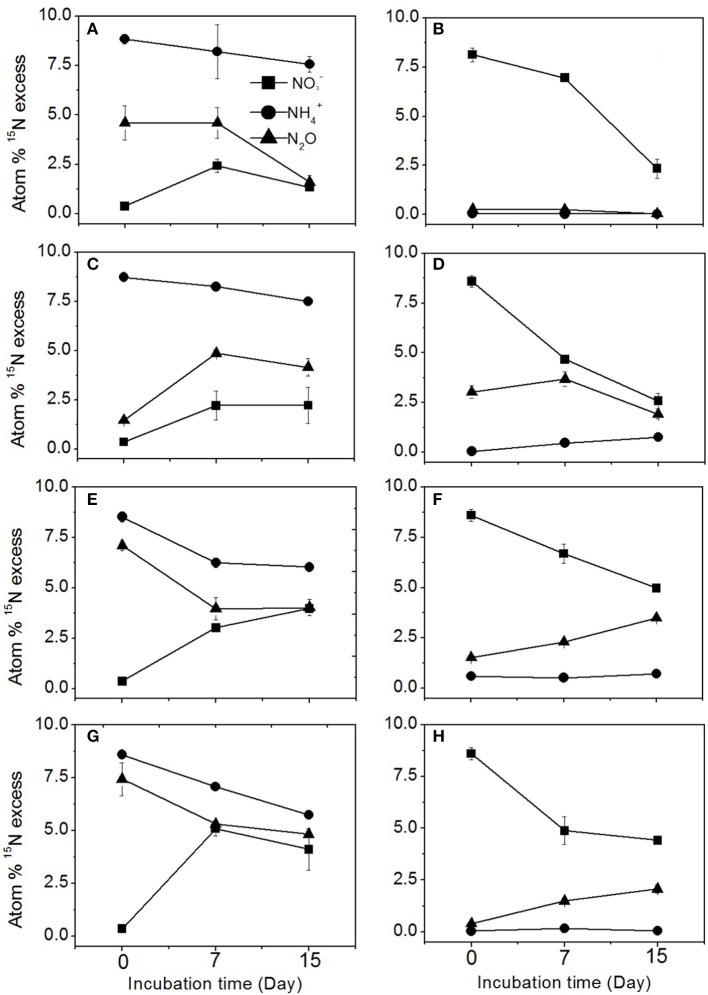
**^15^N enrichment of N_2_O, ${\text{NH}}_{4}^{+}$, and ${\text{NO}}_{3}^{-}$ in the ^15^N labeled ${\text{NH}}_{4}^{+}$ and ${\text{NO}}_{3}^{-}$ treatments during the incubation. (A,C,E,G)** represent the ^15^N-labeled ${\text{NH}}_{4}^{+}$ treatment in the sugarcane, vegetable, dairy pasture, and cereal cropping soils, respectively. **(B,D,F,H)** represent the ^15^N labeled ${\text{NO}}_{3}^{-}$ treatment in the sugarcane, vegetable, dairy pasture, and cereal cropping soils, respectively. Error bars represent standard error.

The enrichment of N_2_O, ${\text{NH}}_{4}^{+}$, and ${\text{NO}}_{3}^{-}$ pool is shown for each treatment in the different agricultural soils in Figure 2. In the ^15^${\text{NH}}_{4}^{+}$ treatment, the ^15^N enrichment in the N_2_O pool over the course of incubation was always between the ^15^N enrichment levels of the ${\text{NH}}_{4}^{+}$ and ${\text{NO}}_{3}^{-}$ pools, suggesting that N_2_O was produced by both nitrification and denitrification (Figures 2A,C,E,G). The denitrification pathway was responsible for only 3.3% of N_2_O production in the sugarcane soil (Table 2), which was reflected by the ^15^N enrichment of N_2_O from the ^15^${\text{NO}}_{3}^{-}$ treatment (Figure 2B). In the sugarcane soil the ^15^N enrichment of N_2_O from the ^15^${\text{NO}}_{3}^{-}$ treatment was close to natural abundance (Figure 2B), and N_2_O was determined to be mainly produced from nitrification (96.7%), which was the same trend as observed for dairy pasture and cereal cropping soils at day 7 (Figures 2F,H). However, in the vegetable soil, the ^15^N enrichment of the N_2_O pool (Figure 2D) was close to the ^15^N abundance of the ^15^${\text{NO}}_{3}^{-}$ at day 7, indicating that denitrification was the predominant pathway of N_2_O emission and was determined to be responsible for 76.3% of N_2_O production (Table 2).

**Table 2:** **Gross nitrification rates and the ratios of N_2_O production to nitrification in the studied agricultural soils**.

**Land-use**	**Gross nitrification rate**	**Relative contribution %**		**N_2_O^c^_d_**	**N_2_O^d^_n_**	**P_N2O_ ‰^e^**
	**mg N kg^−1^ d^−1^**	**C^a^_d_**	**C^b^_n_**	**μg N_2_O-N cm^−2^ d^−1^**		
Sugarcane	1.70 (0.50)	3.30 (0.45)	96.67 (6.8)	0.80 (0.03)	23.40 (0.34)	0.030 (0.0016)
Vegetable	5.42 (0.43)	76.36 (9.2)	23.64 (3.91)	53.65 (7.03)	16.63 (3.30)	0.024 (0.0011)
Dairy Pasture	3.84 (0.78)	29.09 (4.1)	70.90 (4.97)	20.24 (1.22)	49.85 (8.34)	0.033 (0.0026)
Cereal cropping	9.88 (2.30)	28.74 (8.6)	71.26 (1.82)	134.34 (4.06)	334.47 (6.63)	0.260 (0.0189)

a *The relative contribution by denitrification (Cd) to N_2_O production*.

b *The relative contribution by nitrification (Cn) to N_2_O production*.

c *N_2_O production from nitrification (N_2_O_n_)*.

d *N_2_O production from denitrification (N_2_O_d_)*.

e *The proportion of nitrified N emitted as N_2_O (P_N2O_)*.

*Values in bracket are standard deviations*.

The nitrification-derived N_2_O peak from the cereal cropping soil was 334.4 μg N_2_O-N cm^−2^ d^−1^ (Table 2), which was strikingly higher than that in the sugarcane soil (23.4 μg N_2_O-N cm^−2^ d^−1^) although the Cn (the contribution of nitrification to N_2_O production) of the sugarcane soil was higher than that of the cereal cropping soil. In the acidic soils, the Cn was higher than that of denitrification (Cd; Table 2), and followed the order sugarcane soil > cereal cropping soil > dairy pasture soil. There was a negative relationship between the denitrification capacity and soil pH in the three acidic soils (Table 2).

### N_2_O derived from nitrification

The gross nitrification rate was calculated by the ^15^N dilution technique (Barraclough and Puri, 1995), because net nitrification does not necessarily reflect actual scale of processes, particularly where substrate is subject to losses of other pathways. The results showed that the gross nitrification rates were 1.70, 5.42, 3.84, and 9.88 mg N kg^−1^ d^−1^ for the sugarcane, vegetable, dairy pasture, and cereal cropping soils, respectively (Table 2). The nitrification rate in the cereal cropping soil was significantly (*p* < 0.05) higher than that in the other three soils. The proportion of nitrified N emitted as N_2_O (P_N2O_) over 7 days varied across soils (Table 2). The cereal cropping soil had the highest P_N2O_ value (0.26‰) which was significantly (*p* < 0.05) higher than that in other soils. The gross nitrification rates for the four soils followed the order of cereal cropping > vegetable > dairy pasture > sugarcane, whilst P_N2O_ followed different order of cereal cropping > dairy pasture > sugarcane > vegetable.

### Dynamics of ammonia oxidizers during the microcosm incubation

**Figure 3:**
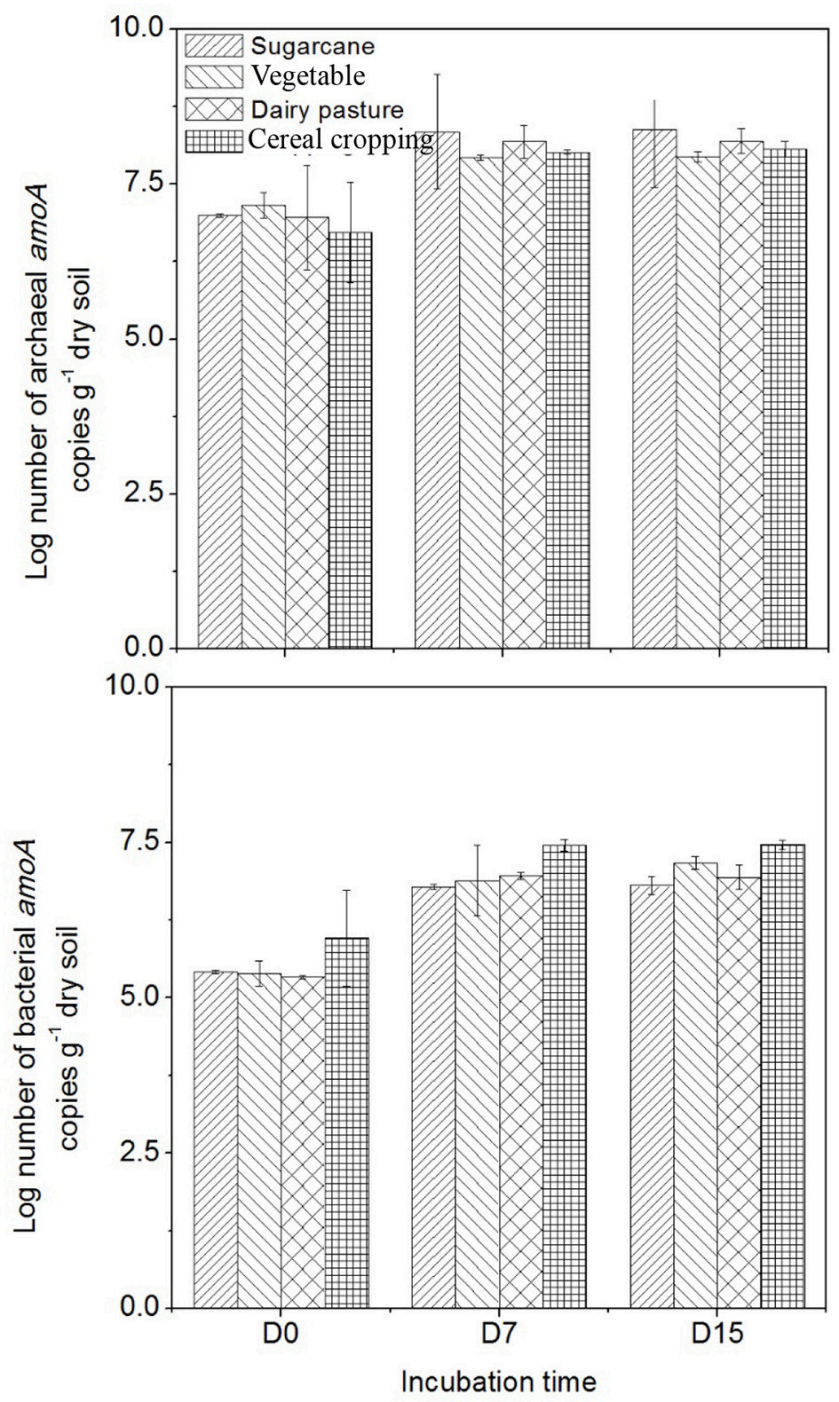
**Changes in AOA and AOB *amo*A gene copy numbers in the ^15^NH_4_ treatment during the incubation period**. Error bars represent standard error.

The abundance of AOB *amo*A genes was always lower than that of AOA *amo*A genes in all four agricultural soils (Figure 3). The highest AOA *amo*A gene abundance was found in the vegetable soil at day 0 (1.5 × 10^7^ copies g^−1^ dry soil), while the highest AOB *amo*A abundance was observed in the cereal cropping soil at day 0 (9.1 × 10^5^ copies g^−1^ dry soil). Following application of fertilizers, both AOA and AOB *amo*A gene abundance significantly increased in the four soils (*p* < 0.05). The cereal cropping soil had the largest AOB population throughout the incubation period (on average 2.9 × 10^7^ copies g^−1^ dry soil), whilst AOA *amo*A gene abundance in the sugarcane soil (on average 2.5 × 10^8^ copies g^−1^ dry soil) were found to be higher than those in the vegetable soil (on average 8.6 × 10^7^ copies g^−1^ dry soil), the cereal cropping soil (on average 1.0 × 10^8^ copies g^−1^ dry soil) and the dairy pasture soil (on average 1.7 × 10^8^ copies g^−1^ dry soil; Figure 3). In the sugarcane soil the ratio of AOA to AOB was the highest and averaged at 61.4, followed by the dairy pasture soil averaged at 24.5, vegetable soil at 23.2 and cereal cropping soil at 5.4 within the whole incubation period. Although AOA were more abundant than AOB, the magnitude of changes in AOB abundance in the microcosm was greater than that of AOA.

### Correlation between nitrification-sourced N_2_O and AOA and AOB populations in different soils

There was a significant correlation between AOA *amo*A gene abundance and nitrification-sourced N_2_O (*p* < 0.05) in sugarcane soil, regardless of the applied labeled fertilizer (Table 3). In contrast, significant relationship (*p* < 0.001) between nitrification-related N_2_O and AOB *amo*A gene abundance was only observed after fertilizer application in cereal cropping soil.

**Table 3:** **Spearman correlations between N_2_O^a^_n_ and the abundances of AOA and AOB**.

**Land-use**	**Factor**	**AOA (log number) *P*-values**	**AOB (log number) *P*-values**
Sugarcane	N_2_O_n_	0.033	0.085
Vegetable	N_2_O_n_	0.038	0.041
Dairy pasture	N_2_O_n_	0.008	0.022
Cereal cropping	N_2_O_n_	0.138	0.0002

a *means N_2_O production from nitrification*.

## Discussion

This study investigated N_2_O production, pathways of soil N_2_O emission, proportion of nitrified N emitted as N_2_O, changes in abundance of ammonia oxidizers, and the correlation between nitrification-related N_2_O and ammonia oxidizer populations in four agricultural soils with different land-use. The results demonstrate that these soils differing in both soil physicochemical properties and land-use have different rates of N_2_O production at a particular WFPS. Although it is not possible to clearly discern the effects of land-use from this experimental design, we speculated that land-use may affect nitrifier-derived N_2_O emissions. Verchot et al. (1999) demonstrated that there were lower N_2_O fluxes from pasture soils compared with forest soils. Studies in the humid and subhumid tropics have indicated that N_2_O fluxes from the fertilized cropping systems can be as much as 10 times that from the natural systems depending on the rates and timing of application of fertilizers (Davidson et al., 1986; Veldkamp and Keller, 1997). This was likely attributed to the different soil properties determined by different land-use. In fact, many previous studies have showed that land-use and management practices could significantly affect soil physical, chemical, and biological parameters (Lauber et al., 2008; Osborne et al., 2011; Bissett et al., 2014).

Firestone and Davidson (1989) indicated that the variable contribution of nitrification and denitrification to N_2_O was mainly due to the varying C and N availability. In this study, the different soils were largely characterized by the differences in soil pH and N, C. It has been reported that soil pH could directly and positively affect denitrification enzymes (Simek and Cooper, 2002), which may be a possible explanation for the higher contribution of denitrification to N_2_O (76%) in the vegetable soil (pH 7.8) compared with the other three acidic soils. Aulakh and Doran (1990) found that most denitrifiers had optimum pH values between 6 and 8 for growth and activity. The contribution of nitrification to N_2_O decreased with decreasing soil pH in the acidic soils (Table 2). This is probably because that acidic soil pH has an overriding effect on autotrophic nitrification and low soil pH can impede the activities of autotrophic nitrifier (Weber and Gainey, 1962). Some studies in subtropical China have revealed that acidic soils reduced nitrification capacity (Zhao et al., 2007). However, the research conducted by Xu and Cai (2007) on 54 denitrification measurements in humid subtropical soils showed that neither the increased pH of upland soil, nor the decreased pH of the tea garden soil altered soil denitrification capacity. The results from Xu and Cai (2007) suggested that land-use and management practices favored soil C and/or N accumulation and anaerobic microorganism activities enhanced soil denitrification capacity.

Furthermore, Weier et al. (1993) demonstrated that total N loss due to denitrification generally increased as soil texture became coarser and without the C-amendment. In this study, the sandy vegetable soil (pH 7.8) which had the lowest organic C content (0.6%) also had a strong denitrification capacity and a high contribution to N_2_O production (76.36%). However, in the sugarcane soil which was also sandy (pH 6.0) and had a low organic C content (0.99%), the contribution of N_2_O production was lowest (around 3.3%) among the four agricultural soils. The possible explanation might be that smaller amounts of organic C and mineral N can be available to the denitrifying population under acidic conditions (Simek and Cooper, 2002). In this study, the highest P_N2O_ (0.26‰) occurred in the cereal cropping soil with the highest organic C indicating that soil organic C content may also have affected N_2_O production ratios from nitrification. Mørkved et al. (2007) found that the ratio of N_2_O production from nitrification in soils with low pH and high organic C content was higher than the soils with high pH and low soil organic C content.

Substrate N level is another important variable influencing N_2_O emissions from soils by affecting the rates and the product spectra of nitrification and denitrification (Moiser, 1994; Kaiser et al., 1996; Skiba et al., 1997). The initial concentration of ${\text{NO}}_{3}^{-}$ in cereal cropping soil was low (10 mg N kg^−1^ soil), but the gross nitrification rate and nitrification-sourced N_2_O ranked the highest among the soil samples after treatments application. The results were in agreement with those obtained by Gödde and Conrad (2000). It may be because nitrifiers limited denitrification by providing ${\text{NO}}_{2}^{-}$ and ${\text{NO}}_{3}^{-}$ which were particularly low in initial concentrations. In our study, the nitrifiers in cereal cropping soil were highly responsive to fertilizer additions leading to the greatest N_2_O emissions from nitrification. The study conducted by Xu and Cai (2007) in the sub-tropical soils inferred that ${\text{NO}}_{3}^{-}$-N concentration was a vital factor affecting denitrification occurrence. Denitrification capacity varied greatly, from nearly absent to complete disappearance of ${\text{NO}}_{3}^{-}$-N added at a rate of 200 mg N kg^−1^ soil within 11 days under anaerobic incubation at 30°C (Xu and Cai, 2007). The results of this study showed the P_N2O_-values were lower than the observations of Zhang et al. (2011).

The different soils had different *amo*A genes copy numbers (Figure 3). Copy numbers of the AOA and AOB *amo*A genes were found to be higher in the sugarcane and cereal cropping soils respectively, than in the other soils, suggesting that *amo*A genes abundances might be influenced by land-use or soil type. Previous studies found similar results when comparing *amo*A genes between different agricultural land-use soils (Hayden et al., 2010; Bissett et al., 2014).

The important role of AOA in nitrification and their potential for N_2_O production has been highlighted previously in different ecosystems (Francis et al., 2005; Könneke et al., 2005; Hu et al., 2015a). Here, it was observed that nitrification-derived N_2_O emissions (Cn) in the cereal cropping soil was significantly correlated to AOB population (*p* < 0.01) while AOA was mainly correlated with nitrification in the sugarcane soil (*p* < 0.05). The sugarcane soil had the lowest amount of substrate (${\text{NH}}_{4}^{+}$), while the ${\text{NH}}_{4}^{+}$ concentration was two times higher in the cereal cropping soil. It has been suggested that AOA prefer by low fertility environments (Di et al., 2009; Schauss et al., 2009), while AOB communities are better adapted to the high nutrient availability conditions (Di et al., 2009). Therefore, in the cereal cropping soil, AOA may only play a minor role in N_2_O production, and AOB were likely to play the predominant role in N_2_O emission. Di et al. (2010) also found that AOB population had a significant relationship with N_2_O production in N-rich grassland soil. We measured the *amo*A gene abundance in different soils based on soil DNA, giving insights into community size and potential contribution to activity, however, measurements of active community based on soil RNA are highly desirable in future studies. Furthermore, community analysis perhaps is also needed to identify the active ammonia oxidizers, in addition to quantifying them. The interpretation of the relative contributions of AOA and AOB to N_2_O emissions cannot be made clearly and the underlying mechanism may need to be studied further using more advanced molecular techniques. Furthermore, it is not possible to accurately determine the relative contribution of AOA and AOB to N_2_O emissions, because the assumptions were made that all AOA and AOB produced the same yield of N_2_O per unit of ammonia oxidized. However, a large body of previous literature stated that this was not the case (Stieglmeier et al., 2014). 1-octyne, a recently reported AOB selective inhibitor, can be used to separate AOA-related N_2_O and AOB-related N_2_O and specifically inhibited AOB growth, activity and N_2_O production (Hink et al., 2016). Therefore, it is essential to make use of AOA or AOB selective inhibitor to give an explicit interpretation on the relative role on nitrification-sourced N_2_O.

## Conclusions

In conclusion, under the experimental aerobic microcosm conditions, nitrification was the main contributor of N_2_O emissions in acidic sugarcane, dairy pasture and cereal cropping soils (pH < 6). Denitrification played a predominant role in N_2_O production in an alkaline vegetable soil. Compared to the cereal cropping, sugarcane, and dairy pasture soils, more nitrification-sourced N_2_O was emitted from the sugarcane soil (Cn 96.67%). AOB might be the major contributor to N_2_O emissions in the cereal cropping soil, while AOA may be predominately responsible for nitrification-sourced N_2_O in sugarcane soil. In the vegetable and dairy pasture soils, both AOA and AOB are likely to contribute to nitrification and N_2_O emissions. Our findings provide evidence that land-use and soil properties may be important factors influencing the contributions of different pathways to N_2_O emissions, and the size of the AOA and AOB communities. Furthermore, further work with more soil samples from similar land-use and/or field trials are required to confirm the laboratory microcosm observations.

## Author contributions

RL made substantial contribution to the content of this article and is the primary author. She was responsible primarily for the planning, execution, and preparation of the work for publication. HS, HwH, HH, JH, PM, and DC contributed substantial time and research funding to help RL to improve the design of the work and the analysis, interpretation of data for the work. They revised it critically for important intellectual content and final approval of the version to be published, also agreement to be accountable for all aspects of the work in ensuring that questions related to the accuracy or integrity of any part of the work are appropriately investigated and resolved.

### Conflict of interest statement

The authors declare that the research was conducted in the absence of any commercial or financial relationships that could be construed as a potential conflict of interest.
